# Exploring the Potential Effects of Soybean By-Product (Hulls) and Enzyme (Beta-Mannanase) on Laying Hens During Peak Production

**DOI:** 10.3390/ani15010098

**Published:** 2025-01-04

**Authors:** Muhammad Shuaib, Abdul Hafeez, Deependra Paneru, Woo Kyun Kim, Muhammad Tahir, Anthony Pokoo-Aikins, Obaid Ullah, Abubakar Sufyan

**Affiliations:** 1 Department of Poultry Science, Faculty of Animal Husbandry & Veterinary Sciences, The University of Agriculture, Peshawar 25000, Pakistan; 2 Arid Zone Small Ruminants Research Institute, Ghulam Banda, Kohat 26010, Pakistan; 3 Department of Poultry Science, University of Georgia, Athens, GA 30602, USA; 4 Department of Animal Nutrition, Faculty of Animal Husbandry and Veterinary Sciences, The University of Agriculture, Peshawar 25130, Pakistan; 5 Toxicology and Mycotoxin Research Unit, USDA-ARS, Athens, GA 30605, USA; 6 Department of Veterinary Clinical Sciences, Faculty of Veterinary and Animal Sciences, University of Poonch, Rawalakot 12350, Pakistan; 7 Department of Livestock and Poultry Production, Bahauddin Zakariya University, Multan 60800, Pakistan

**Keywords:** β-mannanase, digestibility, egg quality, hematology, soybean hulls

## Abstract

This study determined the interaction between soybean hulls (SHs) and enzymes (β-mannanase) to improve the sustainability and efficacy of feeding programs for laying hens during peak production while ensuring the best health and efficiency. In a completely randomized design (CRD), 200 golden-brown hens were fed for four weeks (33 to 36 weeks) and randomly distributed into four groups, each containing four replicates of ten birds, with one group receiving a control diet (P0) and the others receiving diets that contained four combinations of SHs and enzymes (ENZs). e.g., 3% SHs and 0.02 g/kg ENZs (P1), 3% SHs and 0.03 g/kg ENZs (P2), 9% SHs and 0.02 g/kg ENZs (P3), and 9% SHs and 0.03 g/kg ENZs (P4). Although most egg quality measures remained similar, the P2 group showed enhanced (*p* = 0.630) egg weight, albumen weight, and height. Moreover, the P2 group improved gut (*p* < 0.05) shape by increasing villus width, height, crypt depth, and surface area throughout intestinal sections, while the P4 group markedly improved total cholesterol and LDL (*p* = 0.022) levels. The P1, P2, and P4 groups exhibited a significant enhancement in dry matter (*p* = 0.022) and crude fiber (*p* = 0.046) digestibility, while the P2 group demonstrated superior crude protein digestibility (*p* = 0.032), and the P1 and P2 groups showed increased crude fat digestibility compared to the other groups. In conclusion, adding 3% of SHs and 30 mg/kg of ENZs (β-mannanase) to the feed may help laying hens, enhance gut health and some egg quality indices, and decrease blood cholesterol and LDL levels without compromising nutrient digestibility.

## Simple Summary

This study investigated the effect of soybean hulls (SHs) and enzymes (β-mannanase) on the egg quality, serum biochemistry, nutrient digestibility, digesta viscosity, feces consistency, and intestinal histomorphology of golden-brown laying hens during peak production. Different diets containing different levels of SHs and enzymes were fed to the birds for four weeks, and the results showed a significantly improved gut villus width, height, crypt depth, and surface area in the 3% SH and 0.03 g/kg enzyme group than in the remaining groups. This combination also enhanced the dry matter, crude protein, crude fiber, and crude fat digestibility and reduced total cholesterol and LDL levels. Egg quality, digesta viscosity, and feces consistency were not affected. Overall, adding 3% of SHs and 0.03 g/kg of enzymes (β-mannanase) to the feed may help enhance laying hens’ gut health and nutrient digestibility, lowering cholesterol and LDL levels without affecting the egg quality indices.

## 1. Introduction

As the world’s population rises, the poultry business keeps expanding to satisfy consumer demands for animal protein while reducing production costs and environmental effects. Production methods that promote resource conservation, long-term ecological balance, and economic efficiency are part of a sustainable and economical approach to chicken production [1]. Creating poultry diets that meet the nutrient needs of the animals while incorporating substitute feed ingredients like corn distillers, grains, and soybean hulls (SHs) provides alternate sources for the feed-efficient production of food derived from animals for human consumption with notably lower greenhouse gas emissions [2]. Dietary fiber offers a chance for sustainable poultry production in this regard. Poultry performance and health are complicatedly impacted by dietary fiber [3]. Despite being usually regarded as harmless, excessive quantities of insoluble fibers like cellulose and lignin can lengthen the digesta retention period and perhaps interfere with the absorption of nutrients [4]. Despite being good for gut health, soluble fibers like pectin and other hemicelluloses can cause enteric issues at greater doses because they increase the viscosity of digesta [5].

SHs vary in chemical composition due to the efficiency of the dehulling process and, hence, contain varying quantities of celluloses (29–51%), hemicelluloses (10–25%), proteins (11–15%), lignin (1–4%), and pectin (4–8%) [6,7]. In addition to having varying amounts of fiber fractions, SHs are abundant in insoluble fibers, including lignocellulose [8]. Their quick fermentation and the existence of healthy carbohydrates such as galactomannan [9,10,11] offer potential nutritional advantages. Non-starch polysaccharides (NSPs), on the other hand, are also abundant in SHs and can decrease nutrient digestibility, raise digesta viscosity, and inhibit development [12]. β-mannans are a category of heat-resistant compounds found among NSPs that endure the drying–toasting phase during soybean processing and constitute approximately 1.3% to 1.6% of the total content in dehulled and non-dehulled soybeans [13,14,15]. β-mannans are predominantly located in SHs and fiber fractions, exhibiting high viscosity and anti-nutritional properties [16]. The use of SHs in poultry is constrained by insufficient enzymes required to effectively digest the NSPs they contain [17]. Incorporating exogenous enzymes, such as β-mannanase, can effectively tackle this challenge. The enzymes facilitate the breakdown of NSPs, leading to a reduction in digesta viscosity and an enhancement in nutrient digestibility [18]. Therefore, SHs are a more practical and economical feed ingredient since β-mannanase can improve poultry performance and production efficiency.

During the critical peak production stage of hens, the incorporation of SHs and β-mannanase in their diets presents opportunities for enhanced economic and performance outcomes. Soybean hulls, an economic by-product of soybean processing, can partially substitute soybean meal, reducing feed costs while introducing indigestible fibers. The β-mannanase enzyme has the potential to increase egg production without compromising egg quality by effectively breaking down the β-mannans, thereby improving nutrient digestibility. Further research is necessary to determine the ideal ratio of SHs and β-mannanase, as increased concentrations of SHs may necessitate a higher enzyme concentration for optimal results. This presents an opportunity to develop hen diets sustainably and cost-effectively while boosting farm profitability and sustaining or even improving egg production at peak periods.

Therefore, this study examines the influence of various SH and enzyme (β-mannanase) combinations to improve the sustainability and efficacy of feeding programs for laying hens during peak production (33–36 weeks) while ensuring the best health and efficiency.

## 2. Materials and Methods

### 2.1. Experimental Diet, Birds, and Housing

The experiment was approved by the Ethics Committee of the Faculty of Animal Husbandry and Veterinary Sciences (FA&VS), The University of Agriculture Peshawar, Pakistan (No. 6780-A/LM, B&G/UOA, 31 December 2020). We purchased 200 32-week-old golden-brown laying hens (RIR × Fayoumi) (average weight = 1.382 ± 1.06 kg) from a Rawalpindi, Pakistan, commercial market. The hens were fed for four weeks (33 to 36 weeks) and randomly distributed in a completely randomized design (CRD) into five groups (40 birds/group), each containing four replicates of ten birds (five cages/replicate and two birds/cage). The experimental diets (isoenergetic and isoproteic) included a control diet (P0) (lacking SHs and enzymes (ENZs) (β-mannanase) (Hemicell^TM^, Elanco Animal Health, Greenfield, IN, USA) and diets that contained four combinations of SHs and ENZs, e.g., 3% of SHs + 0.02 g/kg of ENZs (P1), 3% of SHs + 0.03 g/kg of ENZs (P2), 9% of SHs + 0.02 g/kg of ENZs (P3), and 9% of SHs + 0.03 g/kg of ENZs (P4), as shown in Table 1. The raw SHs obtained directly from shelling soybeans were used in the formulation of diets, first in a mash form and then pelleted in the SB (Sadiq Brother) feed mill in Rawalpindi, Pakistan. The hens were maintained in the same environmental and management situations. All birds had separate feeders and drinkers with ad libitum water and restricted feed consumption (125 g/day). The house had an ambient temperature of 75 °F and a 17 h/day light period. The birds received vaccinations (ND and IB) as per a standard timetable.

### 2.2. Egg Quality Traits

Four eggs per replication were randomly selected each week for four weeks to assess each egg’s interior and exterior quality. The weight of each egg was determined using a calibrated digital balance (Model: JA-410, Napco, Shenzhen, China). The eggshell’s albumen and yolk were removed and shifted systematically to a Petri dish, and a transparent plastic rod was used to determine the albumen height. A suction method was used to extract the egg yolk from the Petri dish, and the weight of the isolated yolk and albumen was determined using a digital balance. The isolated eggshells were dried overnight at 105 °C in a forced air oven (Model: MA36EG, Success, London, UK) to achieve complete dryness. Then, a micrometer screw gauge (Model: 3001DIG, Baxlo, Barcelona, Spain) was applied to calculate the eggshell (with membranes) thickness at three different sites on each egg (sides and broader and narrow end), and the results were averaged to yield the desired value. Haugh units (HUs) were computed using the formula specified by the authors of [19].

HU = 100log_10_[H + 7.57 − 1.7W^0.37^] (1)

where H = albumen height; W = egg weight.

### 2.3. Hematology and Serum Biochemistry

For hematological and serum biochemical parameters analysis, blood samples (wing veins) from four birds per replicate were taken on the last day of the study. Blood smears stained with Wright–Giemsa were examined under a microscope to identify and quantify the types of white blood cells (WBCs), including Heterophil and Lymphocyte cells. A hemocytometer (Superior™, Marienfeld, Lauda-Königshofen, Germany) was used to perform a manual counting procedure for white blood cell (WBC) and red blood cell (RBC) determination. The microhematocrit method was employed to assess the packed cell volume (PCV) comprised of red blood cells (RBCs). The hemoglobin (Hb) concentration in the blood was measured using the Cyanmethemoglobin methodology [20]. The blood serum was examined for total cholesterol (TC), high-density lipoprotein (HDL), and low-density lipoprotein (LDL) utilizing a commercial kit (Cromatest^®^ Cholesterol MR, Linear Chemicals S.L., Barcelona, Spain). The following formula was used to determine very low-density lipoprotein (VLDL):

VLDL = TC − HDL − LDL(2)

### 2.4. Consistency of Excreta and Digesta Viscosity

To determine digesta viscosity, fresh digesta samples were taken from the gizzard to Meckel’s diverticulum (proximal sample) and from Meckel’s diverticulum to the intersection of the ileum, cecum, and colon (distal samples) using two birds per replication. Once the two samples had been thoroughly combined, approximately 1.5 g (grams) of each were placed into microcentrifuge tubes. After that, the samples were centrifuged at 12,700× *g* for five minutes. The supernatant was then collected, and the viscosity was determined with the assistance of a digital viscometer (Brookfield, MA, USA) at a temperature of 40 °C (degrees Celsius) and a shear rate of 42.5 s^−1^ [21].

The consistency of excreta was visually evaluated and assigned a score during the last week of the trial, utilizing a modified scoring system [22], e.g., normal dry droppings with coning = 1, loose droppings but no free water and some coning = 2, loose droppings with some free water and mild coning = 3, and highly loose droppings with a significant amount of free water and no coning = 4.

### 2.5. Nutrient Digestibility

Nutrient digestibility was determined through the total collection method. Three birds of similar weight were relocated to metabolism cages with feces-catching pans in the last three days of the trial. All diets contained quantified weight of feed with 1% Celite (Celite Corp., Lompoc, CA, USA) as an indigestible marker. Each cage’s excreta were collected twice daily (morning and evening), cleaned to remove feathers and feed constituents, weighed, and kept at 4 °C for analysis. Following AOAC (2000) methods, feed and excreta samples were analyzed for proximate dry matter (DM), crude protein (CP), crude fiber (CF), and ether extract (EE). The following equation describes the index method for assessing nutrient digestibility [23].

ATTD_X_, % = 100 − [(AIA_I_/AIA_O_) × (P_O_/P_I_) × 100] (3)

where ATTD_X_ is the apparent total tract digestibility of nutrients, AIA_I_ is the acid-insoluble ash concentration of feed intake, AIA_O_ is the acid-insoluble ash concentration of excreta output, P_O_ is the nutrient concentration of excreta output, and P_I_ is the nutrient concentration of feed intake.

### 2.6. Intestinal Histomorphology

The mid-duodenum, jejunum, and ileum were collected for gut histomorphology, washed with 10× PBS, and then preserved in 10% buffered formalin for 24 h. Then, the samples were kept in 70% ethanol for 24 h and dehydrated by increasing ethanol concentrations. Next, the samples were cleared in xylene and sectioned to 5 μm thickness in paraffin wax. A compound microscope (Nikon Eclipse 50, Nikon Corporation, Minato City Tokyo, Japan) at the Veterinary Research Institute (VRI), Peshawar, was applied to obtain 200× images of two slices stained with hematoxylin and eosin. Five villi and crypt depth were randomly selected from each replicate to measure their dimension using ImageJ software (version: 1.53u). Villus surface area was determined using the formula [24]:

VSA = 2π × (Average VW ÷ 2) × VH(4)

VSA stands for villus surface area, VW for villus width, and VH for villus height.

### 2.7. Statistical Analysis

All experimental data are shown as means accompanied by the pooled standard error of the mean (SEM). In a completely randomized design (CRD), the general linear model (GLM) method and one-way ANOVA in SPSS 21.0 was used to analyze the data. The differences among the experimental groups were assessed using the Tukey test. Statistical significance was defined as a *p*-value less than 5 (*p* < 0.05).

## 3. Results

### 3.1. Egg Quality Traits

The impact of β-mannanase and SHs on the external quality of eggs is displayed in Table 2. The external egg quality characteristics of the control and treatment groups were not affected (*p* > 0.05). Nevertheless, compared to the P0 group and other SH–enzyme combinations, the P2 group’s egg and eggshell weights were numerically greater. Likewise, no significant changes were seen in eggshell thickness across any of the groups, though the P2 group exhibited a numerically lower (*p* = 0.235) overall average eggshell thickness than the P0 group and other groupings.

Table 3 shows the impact of SHs and β-mannanase in the diet on egg internal quality. There were no changes (*p* > 0.05) in egg yolk or albumen weight between groups, although the control group displayed numerically higher overall yolk weight, while the P2 group had higher overall albumin weight and height than other groups. Similarly, the Haugh unit was unaffected (*p* = 0.699) between groups.

### 3.2. Hematology and Serum Biochemistry

Table 4 displays the results for the serum biochemistry and hematological parameters. All of the groups’ hematological and serum biochemistry indices were within normal limits (*p* > 0.05) and showed no discernible variations. However, TC and LDL were lower (*p* < 0.05) in the SH–enzyme combination groups (P1, P2, P3, and P4) than in the P0 group. The lowest TC levels (*p* = 0.002) were observed in the P4 group. Furthermore, the P2, P3, and P4 groups displayed lower LDL levels (*p* = 0.002) than the P0 and P1 groups.

### 3.3. Nutrient Digestibility, Digesta Viscosity, and Excreta Consistency

Table 5 compares the impact of SHs and β-mannanase on laying hens’ digestibility, viscosity, and excreta consistency. In comparison to the P3 and P0 groups, the P1 and P2 groups had significantly greater dry matter (*p* = 0.002) digestibility. The P2 group had a higher crude protein digestibility (*p* = 0.032) than the P0 and other groups. Crude fiber digestibility was better (*p* = 0.046) in the P2 and P4 groups compared to the P3 and P0 groups. In comparison with the other groups, the P2 group had a greater crude fat digestibility (*p* = 0.019). Ash digestibility did not differ significantly (*p* = 0.108) between any of the groups. Despite no differences (*p* = 0.494) between groups, the P3 group had the highest digesta viscosity. The consistency of the excreta was comparable across all groups with standard dry coning.

In conclusion, P1 and P2 groups appear to be the most advantageous for digestibility, especially for DM, CP, CF, and fat. Reduced amounts of enzymes or larger SHs have less of an impact on digestibility. Although it was not statistically significant, there was a trend towards greater viscosity values with increased SHs and lower amounts of enzymes. Every group’s excreta consistency remained normal, suggesting no negative impacts on gut health.

### 3.4. Intestinal Histomorphology

Table 6 illustrates how the dietary addition of soybean hulls and β-mannanase affects the laying hens’ ileum, jejunum, and duodenum villus morphology. Villus height (Vh) and width (Vw) in the duodenum were greater (*p* = 0.011, *p* = 0.005) in the P2 group than in the P0 group and other combinations. Crypt depth (Cd) followed a similar pattern, with the highest value in the P2 group and significantly lower values in the P0 and P4 groups (*p* = 0.024). Interestingly, the Vh: Cd ratio, an indicator of absorptive surface area, was not affected among the groups (*p* = 0.803). The P2 group had a larger (*p* = 0.002) villus surface area (VSA) than the other groups, indicating enhanced absorptive ability.

In the jejunum, the P2 and P3 groups had greater Vw and Vh (*p* = 0.048, *p* = 0.019) than the control and other combinations. Cd followed a similar pattern again, with the highest value in the P2 group and significantly lower values (*p* = 0.003) in the P0 and P4 groups. In contrast to the duodenum, the Vh: Cd ratio exhibited significant differences (*p* = 0.020) between groups. The P2 group displayed the lowest ratio compared to the P0 group. The VSA trend was the same as Vw and Vh. The P2 group had the highest value and the most significant changes (*p* = 0.014) from the P0 and P4 groups. Similar to the duodenum and jejunum, the ileum’s Vw and Vh patterns were observed, with the P2 group exhibiting the highest significant (*p* = 0.017, *p* = 0.037) variations from the P0 and P4 groups. Cd was again highest in the P2 group and significantly lower (*p* = 0.032) in the P0 and P4 groups. Similar to Vw and Vh, VSA showed a pattern of significant differences (*p* = 0.012) and the greatest value in the P2 group than in the P0 and P4 groups. Overall, the P2 group with SHs and β-mannanase showed the greatest improvement in the intestinal villus shape. This diet maintains the maximum total absorptive surface area and an ideal Vh: Cd ratio while encouraging increased villus height and width.

## 4. Discussion

In laying hens, the later stages of peak egg production, specifically between 32 and 44 weeks of age, introduce distinct difficulties. As hens mature, metabolism slows, leading to a natural reduction in appetite [25]. Hens focus on egg production, directing a substantial amount of their dietary intake towards the synthesis of yolk and albumen, and this leads to a heightened need for crucial nutrients such as protein, calcium, vitamins, and minerals, which intensifies the issue of nutrient shortage, resulting in a vicious cycle. Although dietary fiber is usually thought of as a productivity barrier, it may have a beneficial effect during this crucial period. Moderate fiber (2–4%) promotes the growth of useful gut bacteria, augmenting nutrient absorption, digestion, and immune function [26,27]. Insoluble fiber can extend feeding duration and promote a sense of satiety, which may assist hens in managing their feed intake and avoiding overfeeding [5,26]. Certain fiber sources, such as lignocellulose and inulin, have been shown to increase eggshell thickness and lessen the occurrence of shell fractures and cracks [28,29]. Some fiber sources may stimulate foraging and decrease feather pecking, a well-known laying hen health concern [30].

Different fiber sources have different fermentability and digestible characteristics. High quantities of highly fermentable fibers may decrease the energy available for egg production, even if they are good for gut health [31]. According to recent studies, including SHs containing β-mannanase in diets is a viable strategy for achieving this stability [32,33,34]. As a readily obtainable and economical by-product of treating soybeans, SHs provide the potential to partly substitute soybean meal in poultry diets, hence decreasing feed prices [35]. Nonetheless, the elevated fiber content, mainly consisting of NSPs such as beta-mannans, restricts the digestibility of nutrients and may adversely affect egg production [17]. β-mannanase, an enzyme specialized in the degradation of mannan, serves as a crucial function in this process. By dissolving these complex fibers, β-mannanase improves intestinal nutrient utilization and digestibility [36]. Multiple pieces of research have shown its ability to enhance egg weight, FCR, and egg weight while maintaining egg quality in laying hens [37,38,39]. Defining the ideal synergistic mixture of SHs and β-mannanase is essential for maximizing their effectiveness. Increasing the levels of SHs can lead to further reductions in feed costs; however, it may require an elevated dosage of β-mannanase to ensure effective utilization of nutrients.

Our research examined the effects of feeding laying hens’ various combinations of SHs and the enzyme β-mannanase. Hens fed 3% of SHs and 0.03 g/kg of enzymes showed a numerically greater egg weight, but it was not statistically significant. These results are similar to other studies that showed that adding enzyme supplements to the diet increased the weight of eggs [40,41,42]. Our treatment did not affect eggshell weight, thickness, yolk and albumen weight, albumen height, or Haugh unit. This suggests that the enzyme may have improved nutrient utilization and obtainability but not enough to modify egg constituent parts.

The use of 0.03 g/kg of enzymes and 9% of SHs significantly reduced total cholesterol and LDL levels than the control group. These findings are consistent with earlier research showing that poultry with increased dietary fiber levels had lower blood cholesterol levels [43,44,45]. The fibers found in SHs are both soluble and insoluble. By attaching to cholesterol in the small intestine and blocking its absorption into circulation, the soluble fiber efficiently lowers cholesterol levels and makes it easier for the body to excrete it [46,47]. All of the serum biochemistry and hematology parameters evaluated in our investigation were within the typical limits [48]. The results indicate that the dietary treatments unaffected the laying hens’ internal physiology and general health. Our results are in line with earlier research, showing that adding dietary fiber up to 9% did not significantly alter the poultry’s internal physiology [49,50]. According to this research, moderate quantities of SH-containing enzymes are well-tolerated and do not harm laying hens.

The findings showed that the groups that received 3% of SHs coupled with 0.02 or 0.03 g/kg of β-mannanase had a significantly higher total tract digestibility of DM, ash, CP, CF, and crude fat. This result is in line with other research that found that providing poultry with β-mannanase increased their digestibility [51,52]. This is due to β-mannanase’s capacity to hydrolyze the β-mannans found in SHs, which are notorious for reducing nutritional digestibility by enhancing intestinal digesta viscosity and impairing the action of digestive enzymes [52,53]. Through the breakdown of β-mannans, β-mannanase may efficiently decrease the viscosity of digesta, increase the mixing of enzymes and substrates, and facilitate the absorption of nutrients. The appropriate β-mannanase concentration for digestibility depends on the amount of β-mannans in the diet. Higher levels of β-mannans may need higher β-mannanase concentrations for successful breakdown. Increased SH or decreased β-mannanase levels may negatively impact digestibility, increasing viscosity and reducing nutritional availability. This is in agreement with former research showing decreased digestibility of dry matter, crude protein, crude fiber, and fat in pigs given diets comprising higher quantities of SHs or lower levels of β-mannanase [53]. Augmented digesta viscosity may affect intestinal health and shape, thereby increasing susceptibility to infections and compromising the function of the mucosal barrier [54]. However, in this study, there were no appreciable differences in the viscosity of digesta or consistency of excreta across the groups, suggesting that the quantity of SHs and β-mannanase used had no detrimental effects on gut health.

The findings show that the amalgamation of SHs and β-mannanase works in concert to increase intestinal villus structure and perhaps nutritional absorption. The hens fed a diet of 0.03 g/kg of β-mannanase and 3% of SHs showed the largest villus width and height in all three intestinal regions. These outcomes agree with other findings demonstrating the benefits of moderate fiber and enzyme provision on poultry gut shape [54,55]. β-mannanase precisely marks and destroys NSPs found in SHs, facilitating the release of trapped nutrients and potentially promoting the proliferation of intestinal epithelial cells [56].

## 5. Conclusions

This study’s results indicate that incorporating 3% of SHs along with 0.03 g/kg of β-mannanase could be an effective approach to improve digestibility, promote gut health, and possibly reduce the amount of serum cholesterol in laying hens, all while guaranteeing satisfactory egg traits.

## Figures and Tables

**Table 1:** Experimental diets’ composition (DM-basis).

Nutrient (%)	Diets				
	P0	P1	P2	P3	P4
Soybean Hulls (%)	0	3	3	9	9
β-Mannanase (g/kg)	0	0.02	0.03	0.02	0.03
Corn	53.1	52.1	52.1	50.5	50.5
Canola Meal (CP 34%)	4.15	3.85	3.67	2.16	2.14
Soybean Meal (CP 44%)	24.3	23.6	23.6	22.2	22.2
GM	0	1	1	1	1
PBM High Fat	2	1.02	1	1	0.85
PO	2.79	2.79	2.72	2.67	2.67
NaCl	0.32	0.32	0.41	0.26	0.41
NaHCO_3_	0.10	0.1	0.1	0.1	0.1
CaCO_3_	11.1	10.1	10.3	8.98	9.15
Celite	1	1	1	1	1
DCP	0.78	0.76	0.74	0.76	0.61
DL-M	0.09	0.09	0.09	0.08	0.09
ChCl (70%)	0.09	0.09	0.09	0.09	0.09
Vitamin-P *	0.08	0.08	0.08	0.08	0.08
Mineral-P *	0.05	0.05	0.05	0.05	0.05
Phytase	0.02	0.02	0.02	0.02	0.02
Enro + Etho	0.03	0.03	0.03	0.03	0.03
Total	100	100	100	100	100
Analyzed Value					
DM	89.4	90.5	90.6	91.0	91.1
CP	17.7	17.5	17.5	17.0	17.0
CF	2.85	2.87	2.87	2.90	2.90
Fat	4.82	4.76	4.77	4.72	4.72
Ash	13.5	13.7	13.7	13.3	13.4
Moisture	10.5	9.48	9.38	9.00	8.84
NFE	51.6	49.0	49.0	45.8	45.8

* To provide one kg of diet: retinyl acetate 4400 IU; DL-α-tocopheryl acetate 12 IU; cholecalciferol: 118 µg; thiamine 2.5 mg; menadione sodium bisulfite 2.40 mg; niacin 30 mg; Vit. B2 4.8 mg; D-pantothenic acid 10 mg; Vit. B6 5 mg; Vit. B7 130 µg; cyanocobalamin 19 µg; Vit. B9 2.5 mg; Mn 85 mg; zinc 75 mg; Fe 80 mg; iodine 1 mg; selenium 130 µg; copper 6 mg. Guar meal (GM); poultry by-product meal (PBM); poultry oil (PO); sodium chloride (NaCl); sodium bicarbonate (NaHCO_3_); calcium carbonate (CaCO3); dicalcium phosphate (DCP); choline chloride (ChCl); vitamin-premix (Vitamin-P); mineral premix (Mineral-P); enramycin + ethoxyquin (Enro + Etho); DL-methionine (DLM); dry matter (DM); crude protein (CP); crude fiber (CF); nitrogen-free extract (NFE). Control = P0; P1 = 3% of SHs and 0.02 g/kg of enzymes (ENZs); P2 = 3% of SHs and 0.03 g/kg of ENZs; P3 = 9% of SHs and 0.02 g/kg of ENZs; P4 = 9% of SHs and 0.03 g/kg of ENZs (β-mannanase).

**Table 2:** Impact of soybean hulls and β-mannanase in the diet on external egg quality.

Parameter	Week	Diets ^1^						
		P0	P1	P2	P3	P4	SEM	*p*-Value
Egg weight (g)	33	55.50	56.00	56.70	55.70	56.20	0.55	0.591
	34	56.00	56.50	56.70	56.00	56.50	0.62	0.914
	35	55.70	56.00	56.20	56.00	56.20	0.65	0.983
	36	56.20	56.50	56.70	56.50	56.00	0.81	0.974
	Overall	55.80	56.20	56.60	56.00	56.20	0.32	0.630
Eggshell weight (g)	33	4.99	5.04	5.10	5.01	5.06	0.04	0.591
	34	5.32	5.36	5.39	5.32	5.36	0.05	0.914
	35	5.12	5.18	5.34	5.16	5.27	0.07	0.414
	36	5.45	5.48	5.50	5.48	5.43	0.07	0.974
	Overall	5.22	5.26	5.33	5.24	5.28	0.03	0.284
Eggshell thickness (mm)	33	0.31	0.30	0.28	0.31	0.31	0.03	0.475
	34	0.30	0.31	0.31	0.32	0.31	0.02	0.939
	35	0.35	0.33	0.28	0.32	0.31	0.04	0.150
	36	0.33	0.33	0.33	0.33	0.36	0.13	0.446
	Overall	0.32	0.32	0.30	0.32	0.32	0.04	0.235

^1^ P0 = Control; P1 = 3% of soybean hulls (SHs) and 0.02 g/kg of enzymes (ENZs); P2 = 3% of SHs and 0.03 g/kg of ENZs; P3 = 9% of SHs and 0.02 g/kg of ENZs; P4 = 9% of SHs and 0.03 g/kg of ENZs (β-mannanase); standard error mean (SEM).

**Table 3:** The impact of soybean hulls and β-mannanase in the diet on internal egg quality.

Parameter	Week	Diet ^1^						
		P0	P1	P2	P3	P4	SEM	*p*-Value
Yolk weight (g)	33	16.80	16.50	16.70	17.00	17.20	0.23	0.660
	34	16.80	15.80	15.90	16.10	16.40	0.23	0.155
	35	16.60	16.40	16.30	16.80	16.70	0.23	0.721
	36	16.50	16.30	16.30	16.70	16.10	0.20	0.428
	Overall	16.70	16.20	16.30	16.60	16.60	0.12	0.179
Albumen weight (g)	33	32.80	33.60	34.00	32.90	33.10	0.36	0.216
	34	33.00	34.40	34.60	33.70	33.80	0.35	0.065
	35	33.10	33.60	33.70	33.10	33.40	0.41	0.849
	36	33.40	33.90	34.00	33.40	33.60	0.53	0.925
	Overall	33.10	33.80	34.10	33.30	33.50	0.20	0.074
Albumen height (mm)	33	6.72	6.85	6.97	6.60	6.75	0.18	0.730
	34	6.75	6.90	6.97	6.87	7.00	0.22	0.953
	35	6.57	6.72	6.77	6.60	6.65	0.19	0.956
	36	6.67	6.77	6.82	6.70	6.77	0.20	0.987
	Overall	6.68	6.81	6.88	6.69	6.79	0.07	0.345
Haugh Unit	33	73.50	75.50	76.80	74.30	74.60	1.57	0.677
	34	71.11	73.20	74.50	71.90	72.20	1.65	0.678
	35	74.13	76.10	76.70	75.20	75.70	1.86	0.887
	36	74.90	76.90	77.50	76.00	76.50	1.83	0.879
	Overall	73.40	75.40	76.40	74.40	74.80	1.47	0.699

^1^ P0 = Control; P1 = 3% of soybean hulls (SHs) and 0.02 g/kg of enzymes (ENZs); P2 = 3% of SHs and 0.03 g/kg of ENZs; P3 = 9% of SHs and 0.02 g/kg of ENZs; P4 = 9% of SHs and 0.03 g/kg of ENZs (β-mannanase); standard error mean (SEM).

**Table 4:** The impact of soybean hulls and β-mannanase on laying hens’ hematological and serum biochemical indicators.

	Diet ^1^						
Hematological Indices ^2^	P0	P1	P2	P3	P4	SEM	*p*-Value
Red blood cells (10^6^/μL)	3.05	3.16	3.17	3.09	3.11	0.04	0.556
White blood cells (10^3^/μL)	3.05	2.98	2.96	3.03	3.02	0.01	0.060
Heterophil (%)	23.00	23.20	24.70	22.70	23.70	1.39	0.885
Lymphocyte (%)	59.00	61.20	62.00	60.50	61.00	1.30	0.598
Heterophil: Lymphocyte	0.39	0.37	0.39	0.38	0.38	0.02	0.978
Packed cell volume (%)	30.00	31.20	31.40	30.30	31.00	0.65	0.413
Hb (g/dL)	9.52	9.78	9.82	9.64	9.71	0.05	0.064
MCHC (g/dL)	32.00	31.30	31.20	32.00	31.30	0.67	0.561
MCV (fL)	98.30	98.70	99.00	98.00	100.00	2.69	0.947
**Serum Biochemical Indices ^3^**							
TC (mg/dL)	130.00 ^a^	127.00 ^b^	126.00 ^b^	124.00 ^bc^	122.00 ^c^	1.39	0.022
HDL (mg/dL)	65.00	65.40	67.90	64.10	66.00	1.31	0.446
LDL (mg/dL)	42.00 ^a^	39.40 ^ab^	37.30 ^bc^	36.00 ^c^	35.00 ^c^	0.99	0.002
VLDL (mg/dL)	23.00	21.20	20.80	23.60	21.20	1.58	0.676
Total protein (mg/dL)	4.94	5.02	5.08	4.98	5.05	0.05	0.508

Various superscripts on the mean in the same row show a significant effect (*p* < 0.05). ^1^ P0 = Control; P1 = 3% of soybean hulls (SHs) and 0.02 g/kg of enzyme (ENZs); P2 = 3% of SHs and 0.03 g/kg of ENZs; P3 = 9% of SHs and 0.02 g/kg of ENZs; P4 = 9% of SHs and 0.03 g/kg of ENZs (β-mannanase); standard error mean (SEM). ^2^ Hb = hemoglobin; MCHC = mean corpuscular hemoglobin concentrations; MCV = mean corpuscular volume. ^3^ TC = Total cholesterol; HDL = high density lipoprotein; LDL = low density lipoprotein; VLDL = very low-density lipoprotein.

**Table 5:** Impact of dietary supplementation of soybean hulls and β-mannanase on nutrient digestibility, digesta viscosity, and excreta consistency in laying hens.

Nutrient (%)	Diet ^1^						
	P0	P1	P2	P3	P4	SEM	*p*-Value
Dry matter, %	78.10 ^b^	81.00 ^a^	82.00 ^a^	79.00 ^b^	80.00 ^ab^	0.73	0.022
Ash, %	57.00	59.20	59.40	58.00	58.20	0.83	0.108
Crude protein, %	67.00 ^c^	69.00 ^b^	72.00 ^a^	67.30 ^c^	68.00 ^bc^	1.11	0.032
Crude fiber, %	68.10 ^b^	70.40 ^a^	71.30 ^a^	68.20 ^b^	70.00 ^a^	0.95	0.046
Crude fat, %	75.00 ^d^	78.00 ^ab^	80.00 ^a^	76.00 ^cd^	77.00 ^bc^	0.98	0.019
Viscosity, (cP) ^2^	4.77	4.72	4.67	4.98	4.82	0.102	0.494
Excreta consistency ^3^	1	1	1	1	1	N/A	N/A

Various superscripts on the mean in the same row show a significant effect (*p* < 0.05). ^1^ P0 = Control; P1 = 3% of soybean hulls (SHs) and 0.02 g/kg of enzymes (ENZs); P2 = 3% of SHs and 0.03 g/kg of ENZs; P3 = 9% of SHs and 0.02 g/kg of ENZs; P4 = 9% of SHs and 0.03 g/kg of ENZs (β-mannanase); standard error mean (SEM). ^2^ cP = Digesta viscosity measured in centipoise. ^3^ Excreta consistency score was ‘1’ in all groups, showing normal dry droppings and coning.

**Table 6:** Impact of soybean hulls and β-mannanase on the nutrient’s digestibility, digesta viscosity, and excreta consistency.

Parameters		Diet ^1^						
		P0	P1	P2	P3	P4	SEM	*p*-Value
Duodenum ^2^	VW (µm)	77.00 ^c^	87.00 ^b^	94.00 ^a^	82.00 ^bc^	87.00 ^b^	2.65	0.005
	Vh (µm)	589.00 ^d^	610.00 ^b^	617.00 ^a^	602.00 ^c^	611.00 ^b^	6.71	0.011
	Cd (µm)	109.00 ^c^	116.00 ^b^	122.00 ^a^	110.00 ^c^	114.00 ^b^	4.15	0.024
	Vh: Cd	5.41	5.28	5.06	5.47	5.38	0.25	0.803
	VSA (mm^2^)	0.14 ^d^	0.16 ^b^	0.18 ^a^	0.15 ^c^	0.16 ^b^	0.04	0.002
Jejunum	Vw (µm)	65.00 ^c^	70.00 ^b^	75.00 ^a^	69.00 ^b^	73.00 ^a^	2.26	0.048
	Vh (µm)	441.00 ^d^	457.00 ^b^	465.00 ^a^	451.00 ^c^	458.00 ^b^	6.84	0.019
	Cd (µm)	60.00 ^d^	68.00 ^b^	76.00 ^a^	64.00 ^c^	71.00 ^b^	2.45	0.003
	Vh: Cd	7.45 ^a^	6.69 ^ab^	6.16 ^b^	7.09 ^ab^	6.46 ^ab^	0.23	0.020
	VSA (mm^2^)	0.09 ^d^	0.10 ^b^	0.11 ^a^	0.09 ^c^	0.10 ^b^	0.05	0.014
Ileum	Vw (µm)	59.00 ^c^	63.00 ^b^	66.00 ^a^	60.00 ^c^	64.00 ^b^	2.22	0.017
	Vh (µm)	389.00 ^c^	397.00 ^b^	403.00 ^a^	386.00 ^c^	394.00 ^b^	5.89	0.037
	Cd (µm)	55.00 ^c^	57.00 ^bc^	61.00 ^a^	56.00 ^c^	58.00 ^b^	1.91	0.032
	Vh: Cd	7.10	6.98	6.67	6.92	6.81	0.21	0.714
	VSA (mm^2^)	0.07 ^c^	0.08 ^b^	0.08 ^a^	0.07 ^c^	0.08 ^b^	0.04	0.012

Various superscripts on the mean in the same row show a significant effect (*p* < 0.05). ^1^ P0 = Control; P1 = 3% of soybean hulls (SHs) and 0.02 g/kg of enzymes (ENZs); P2 = 3% of SHs and 0.03 g/kg of ENZs; P3 = 9% of SHs and 0.02 g/kg of ENZs; P4 = 9% of SHs and 0.03 g/kg of ENZs (β-mannanase); standard error mean (SEM). ^2^ Vw = villus width; Vh = villus height; Cd = crypt depth; Vh: Cd = villus height to crypt depth ratio; VSA = villus surface area.

## Data Availability

The corresponding author may provide supporting data for this research upon reasonable demand.
